# The Concept of α-Synuclein Strains and How Different Conformations May Explain Distinct Neurodegenerative Disorders

**DOI:** 10.3389/fneur.2021.737195

**Published:** 2021-10-05

**Authors:** Katja Malfertheiner, Nadia Stefanova, Antonio Heras-Garvin

**Affiliations:** Laboratory for Translational Neurodegeneration Research, Department of Neurology, Medical University of Innsbruck, Innsbruck, Austria

**Keywords:** α-synucleinopathies, experimental models, biomarker, α-synuclein, strains

## Abstract

In the past few years, an increasing amount of studies primarily based on experimental models have investigated the existence of distinct α-synuclein strains and their different pathological effects. This novel concept could shed light on the heterogeneous nature of α-synucleinopathies, a group of disorders that includes Parkinson's disease, dementia with Lewy bodies and multiple system atrophy, which share as their key-molecular hallmark the abnormal aggregation of α-synuclein, a process that seems pivotal in disease pathogenesis according to experimental observations. However, the etiology of α-synucleinopathies and the initial events leading to the formation of α-synuclein aggregates remains elusive. Hence, the hypothesis that structurally distinct fibrillary assemblies of α-synuclein could have a causative role in the different disease phenotypes and explain, at least to some extent, their specific neurodegenerative, disease progression, and clinical presentation patterns is very appealing. Moreover, the presence of different α-synuclein strains might represent a potential biomarker for the diagnosis of these neurodegenerative disorders. In this regard, the recent use of super resolution techniques and protein aggregation assays has offered the possibility, on the one hand, to elucidate the conformation of α-synuclein pathogenic strains and, on the other hand, to cyclically amplify to detectable levels low amounts of α-synuclein strains in blood, cerebrospinal fluid and peripheral tissue from patients. Thus, the inclusion of these techniques could facilitate the differentiation between α-synucleinopathies, even at early stages, which is crucial for successful therapeutic intervention. This mini-review summarizes the current knowledge on α-synuclein strains and discusses its possible applications and potential benefits.

## Introduction

Alpha-synuclein (α-syn) is an abundant neuronal protein that is primarily found in the presynaptic nerve terminals (1). Although the exact functions of α-syn remain under discussion, different studies have proposed its involvement in the regulation of neurotransmitter release, synaptic function and plasticity, and several cellular processes such as mitochondrial function, gene regulation and its participation in SNARE complex assembly (2). However, under pathological conditions α-syn accumulates and forms insoluble aggregates, which constitute the main feature of a group of neurodegenerative disorders referred to as α-synucleinopathies (2). Thus, the presence of these proteinaceous, α-syn-rich inclusions in neurons or glial cells has been linked to the different disease patterns of Parkinson's disease (PD), dementia with Lewy bodies (DLB), multiple system atrophy (MSA).

In PD and DLB α-syn inclusions, denominated Lewy bodies (LBs), are predominantly present in neurons and neurites (3). Both are age-related disorders influenced by genetic and environmental factors (4, 5). In PD, the most common disease out of all α-synucleinopathies, the occurrence of LBs is associated with the loss of dopaminergic neurons in the substantia nigra, resulting in the prevalent motor symptoms including bradykinesia, resting tremor, muscle rigidity and postural instability (6). Besides the accumulation of α-syn, further hallmarks of PD include lysosomal and proteasomal impairment (7, 8), mitochondrial dysfunction (9), increased iron levels (10) and neuroinflammation (11). As for DLB, although it is also characterized by the presence of Lewy bodies, it differentiates from PD by the predominance of dementia within the first year of Parkinsonism (12). Moreover, while the neuronal loss in PD is mostly affecting the brainstem and the limbic regions, in DLB it predominates in the neocortex with most prevalent symptoms including fluctuating cognition, recurrent visual hallucinations and spontaneous extrapyramidal motor features (13, 14).

In contrast to DLB and PD, MSA possesses fundamental differences in its pathological and clinical presentation. The most pivotal being the predominant presence of α-syn inclusions in the cytoplasm of oligodendrocytes termed glial cytoplasmic inclusions (GCIs) (15) and selective neurodegeneration in multiple brain areas including substantia nigra, striatum, cerebellum, pontine nuclei, and spinal cord, resulting in parkinsonism, cerebellar ataxia and autonomic failure (16). Other important neuropathological features of MSA are neuroinflammation (17, 18), with microglial activation (19), astrogliosis (20) and T-cell infiltration (21). In contrast to the other disorders, MSA usually presents a rapid progression leading to death few years after symptoms onset (22, 23). In addition, despite the existence of some multiplex families, MSA is currently considered a sporadic disease, with no clear signs of genetic involvement in its etiology (24).

Together with the classical α-synucleinopathies (PD, DLB and MSA), different studies have recently included pure autonomic failure (PAF) and isolated rapid eye movement sleep behavior disorder (iRBD) in the group of α-synucleinopathies (25, 26). PAF is a neurodegenerative disorder affecting the autonomic nervous system characterized by orthostatic hypotension, fixed puls rate, anhidrosis, erectile dysfunction and constipation without motor features (27). Similar to the other α-synucleinopathies, one of the main pathological features of PAF is the presence of α-syn inclusions, mostly found in autonomic ganglia and nerves of the peripheral nervous system that is associated with the loss of sympathetic ganglia and postganglionic fibers (27). However, autonomic abnormalities including orthostatic hypotension are also common in classical α-synucleinopathies. In this regard, there are documented cases of patients with PAF phenotype progressing into MSA, DLB and PD, suggesting that it could constitute a prodromal stage of the latter (28–30). In addition, iRBD constitutes a common prodromal syndrome in classical α-synucleinopathies that is associated with the loss of neurons in brainstem areas involved in the regulation of sleep (31–33). Thus, with more than 80% of patients progressing into PD, DLB or MSA (34, 35), iRBD is currently considered an early stage α-synucleinopathy (26).

Interestingly, although α-synucleinopathies all share the presence of misfolded α-syn deposition, they present a wide spectrum of clinical phenotypes with multiple overlapping features. In this regard, data from experimental models and clinical studies have suggested that the abnormal accumulation of α-syn in different cell types and brain areas might be explained by the existence of distinct α-syn strains that could also lead to the different neurodegenerative and disease duration patterns observed in these disorders. This mini-review discusses the current knowledge about the concept of α-syn strains and how different conformations may explain distinct neurodegenerative disorders.

### α-syn: Structure and Aggregation Mechanism

α-syn is composed of 140 amino acids and comprises three distinct domains: the acidic C-terminal region, the highly hydrophobic central portion also referred to as the “non-amyloid component” (NAC), and the N-terminal repeat region (1). The structure of native brain α-syn is still under discussion. It was long considered an intrinsically disordered protein believed to exist in cells as an unstructured monomer (36, 37) until new studies also suggested an α-helical structure of the protein forming tetramers (38, 39). Therefore, a remarkable conformational plasticity is attributed to α-syn, suggesting that native α-syn exists in equilibrium between different conformational states within the cell (1).

Due to its hydrophobic composition, the central NAC region allows the oligomerization of α-syn by undergoing a conformational change to β-sheet structure (40–42). Pathological α-syn then self-assembles into protofibrils which can further form compact amyloid fibrils enriched in β-sheet structure (43). Although there also appear to be long-range interactions between C-terminus and NAC region, and between C- and N-terminus, they are thought to prevent aggregation of native α-syn under physiological conditions (44).

The exact mechanism leading to the aggregation of α-syn under pathological conditions is still unclear. Preclinical studies pointed out that several factors such as oxidative stress, metal ions, proteolysis, post-translational modifications or the presence of fatty acids, among others, can induce or modulate α-syn aggregation and be able to influence the equilibrium between the monomeric and oligomeric state *in vivo* (45). Furthermore, missense and copy number mutations in the *SNCA* gene encoding α-syn which are found in rare familial forms of α-synucleinopathies are also linked to the conformation and aggregation characteristics of α-syn. Interestingly, while some mutations increase the aggregation propensity of α-syn, others have the opposite result (46). However, the majority of α-synucleinopathy cases occur sporadically without the involvement of mutations. Therefore, the mechanism underlying α-syn aggregation in α-synucleinopathies remains elusive and seems to be the result of a multifactorial process that might include different intracellular, epigenetic and environmental factors (2).

### α-syn Spreading and Neurodegeneration: Lessons From Experimental Models

As mentioned before, in homeostatic conditions α-syn is mainly located in the presynaptic terminals. Thus, the source of α-syn in LBs of PD and DLB patients are presumably the neurons themselves. In contrast, in MSA the origin of α-syn in oligodendroglial inclusions is still under discussion (15). In this regard, two possible sources of α-syn have been hypothesized: i) neurons, which are thought to transmit α-syn through a yet unknown mechanism, ii) and oligodendrocytes themselves. The latter constitute also a matter of debate since some studies claim there is no α-syn expression in oligodendrocytes (47, 48), while others revealed even an increased expression in MSA oligodendrocytes compared to healthy controls (49). Regarding the possible neuronal origin of pathological α-syn in MSA, there is supporting experimental data that demonstrate the ability of α-syn to be transferred from neuron to neuron and from neuron to oligodendrocytes (50, 51). Clinically, the first evidence supporting the transmission of α-syn in humans was the presence of LBs in grafted neurons in host brains of PD patients (52, 53). This event also raised the idea that pathogenic α-syn might follow the prion replication paradigm, in terms of propagation and infection mechanism, by recruiting endogenous wildtype monomeric α-syn to form amyloid fibrils (45).

Different experimental models have been generated over the years to study the pathogenesis of α-synucleinopathies and the potential of α-syn to spread throughout the central nervous system (CNS). In this regard, cellular models are not limited to the investigation of the underlying mechanisms of α-syn toxicity and aggregation but have also been used to study the prion-like transmission of α-syn. In particular, *in vitro* α-syn overexpression in cell lines and primary cultures has allowed researchers to study α-syn aggregation and its intercellular trafficking in different cellular environments that reflect the cellular milieu in which LBs or GCIs are formed (54). Consequently, pathological α-syn has been associated with the impairment of several cellular mechanisms such as mitochondrial function, lysosomal and proteasomal activity, axonal transport and Ca2+ homeostasis (2). Although cellular models are useful to study basic pathological mechanisms they fail to portrait them within the framework of a complex organism.

The use of *in vivo* models has also contributed to clarify the pathological consequences of α-syn accumulation and its role in the neurodegenerative processes underlying α-synucleinopathies. In this regard, genetically modified animals overexpressing wild-type (wt) or mutant forms of human α-syn have been extensively used in the last two decades (55–57). Thus, by specifically overexpressing α-syn in different cell types within the CNS or particular brain regions, several research groups were able to mimic different α-synucleinopathies (55–57). The first attempt to model α-synucleinopathies was made by overexpressing human α-syn under the non-specific PDGFβ gene promoter resulting in amorphous aggregates rather than α-syn fibrils as in LBs (57). Expression of mutant forms of human α-syn more adequately reflected the ability of α-syn to induce pathological changes including age-related progressive neurodegeneration with α-synuclein aggregation, astrocytosis, spinal motoneuron loss and paralysis of limbs (57). Several attempts were made to specifically overexpress α-syn in the dopaminergic neurons, one of the main cell types affected by neurodegeneration in PD (57). Unfortunately, this approach was inefficient in modeling the disease adequately. Nevertheless, specific α-syn overexpression under oligodendroglial promoters partially recapitulated in animal models the inclusion morphology, neurodegeneration, neuroinflammation, oxidative stress, motor impairment and non-motor symptoms observed in MSA patients (56). Despite all these observations, and although transgenic models provide an important insight into the effect of α-syn pathology *in vivo*, they still hold a major drawback in modeling disease initiation. By one hand, these animals are based on the artificial overexpression of α-syn, thus masking the original events that might trigger α-syn pathology in patients. On the other hand the overexpression of α-syn leads to α-syn protein levels exceeding the ones associated with idiopathic α-synucleinopathies (58).

The third approach to model α-synucleinopathies involves the inoculation of recombinant α-syn preformed fibrils (PFFs). This method has been especially important in α-syn propagation studies and relies on the property of α-syn to seed and recruit endogenous α-syn, leading to its aggregation and to the spreading of the pathology both *in vitro* and *in vivo* (59, 60). Intracerebral injections of PFFs into the substantia nigra of mice provoked α-syn pathology in its projection areas such as striatum and amygdala and in the stria terminalis receiving amygdala projections (61). Similarly, in striatum-inoculated mice α-syn spreading was induced in interconnected brain regions resulting in progressive loss of dopaminergic neurons in the substantia nigra leading to reduced dopamine levels and motor deficits (62). Moreover, PFFs were also injected into the olfactory bulb to investigate early olfactory dysfunction observed in PD patients, which resulted in the spreading of the pathology into widespread areas including substantia nigra but with no signs of motor dysfunction (63). Besides intracerebral injections, recent studies have also shown that α-syn pathology can spread into the CNS after peripheral inoculation and that different injection sites might elicit distinct propagation patterns (64–68). Thus, inoculation of PFFs in the gastrointestinal tract of rodents induced α-syn pathology that spread from the gut to the brain through the vagus nerve, inducing PD-like motor and non-motor symptoms (66, 67), while the injection of PFFs in autonomic ganglia resulted in a model of PAF (68).

**Figure 1:**
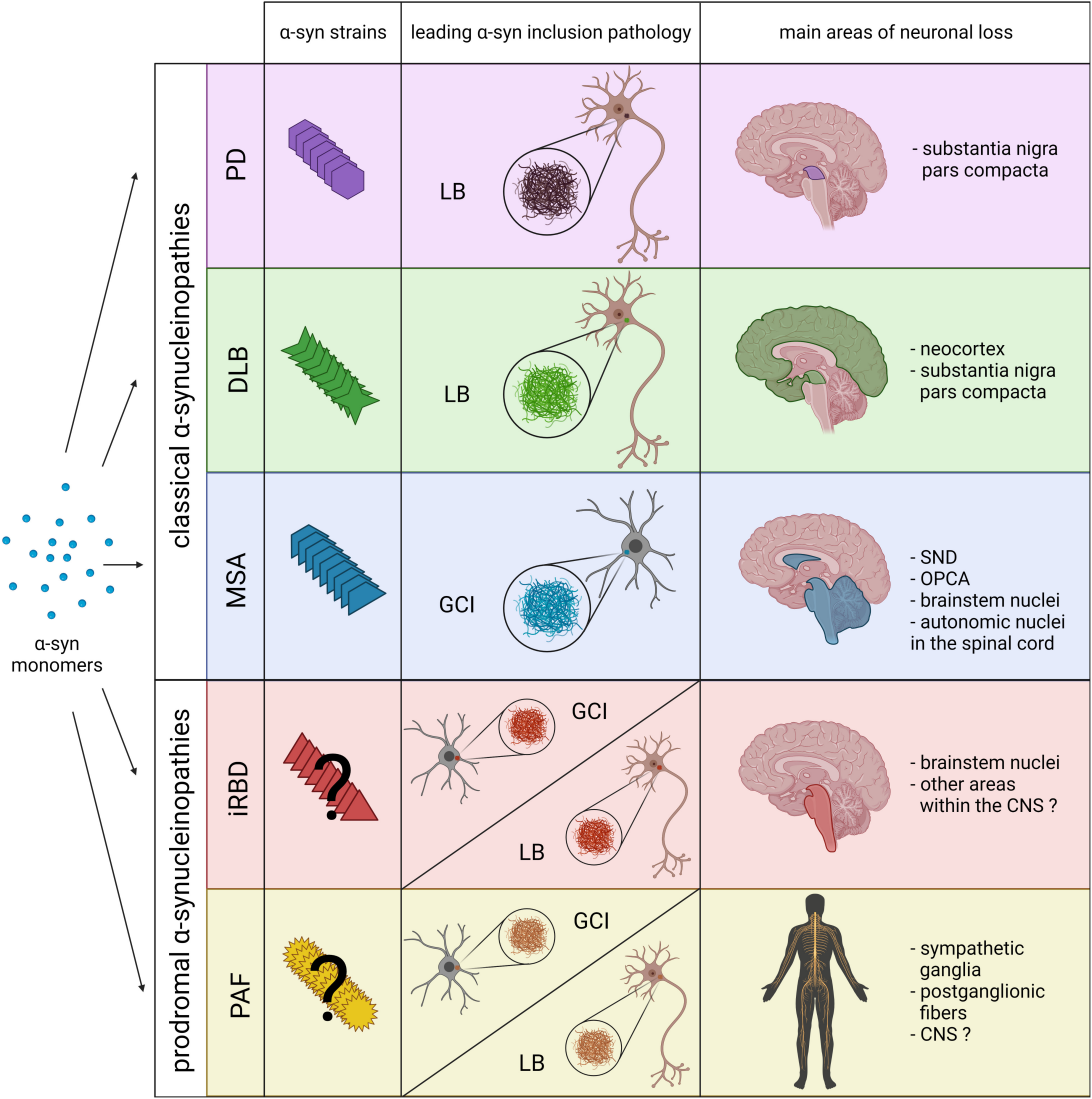
Schematic overview including the main pathological features of prodromal and classical α-synucleinopathies. The combination of novel structural and biochemical analyses have demonstrated the existence of different α-syn strains in classical α-synucleinopathies (PD, DLB and MSA). Recent experimental data also indicate that distinct α-syn strains might be associated, not only with the affected cell type and type of α-syn inclusion (neuronal LBs vs. oligodendroglial GCIs), but also with the neurodegeneration pattern and disease severity. However, at present it is still unclear whether distinct α-syn strains are also associated with prodromal α-synucleinopathies, or if they only constitute early phases of classical α-synucleinopathies. Thus, pathological α-syn in PAF and iRBD patients could lead to the formation of LBs, GCIs and the extension of neuronal loss to other areas within the CNS. SND, striatonigral degeneration; OPCA, olivopontocerebellar atrophy. Created with BioRender.com.

In addition to the injection site, different types of PFFs or “strains” are able to induce distinguishable features. In 2013, by using different buffer conditions, Bousset and colleagues generated two structurally distinct α-syn conformers, denominated “ribbons” and “fibrils” that possessed different levels of toxicity, seeding and propagation properties, with (69). Subsequent *in vivo* experiments further confirmed the capability of both confomers to amplify in a strain-specific manner, presenting differential seeding capacities and inducing strain-specific pathologies and neurotoxic phenotypes (70). Thus, fibrils showed higher toxicity, which was associated with higher oxidative stress, synaptic impairment, neurodegeneration and proteinase K resistance (69, 70). Interestingly, despite a less toxic potential, ribbons induced the formation of more inclusions containing phosphorylated α-syn than fibrils and were also able to generate α-syn positive inclusions within oligodendrocytes, one of the main pathological hallmarks of MSA (70). Additional studies have also demonstrated the ability of recombinant α-syn to generate different strains under different salt conditions, which showed different disease incubation periods, region-specific α-syn deposition patterns and unique clinical signs of neurological illness when injected in transgenic mice (71). In consideration of these findings, it has been hypothesized that different α-syn strains might underlie disease heterogeneity in α-synucleinopathies (Figure 1).

### Patient-Derived α-syn Strains

In recent years, increasing evidence supports the prion-like behavior of proteins accumulating in different neurodegenerative disorders, including α-syn. Prion-like features were first attributed to α-syn due to its ability to induce the aggregation of native α-syn into fibrillary structures forming pathogenic inclusions (45) and, although no evidences of infectivity across individuals has been found in α-synucleinopathies, several studies have shown the existence of different strains or conformations (2) (Table 1).

**Table 1:** Evidences supporting the existence of distinct strains in classical α-synucleinopathies and limitations of the current studies.

	**Current knowledge**	**Limitations of current studies**
Toxicity in vitro/in vivo	PD, DLB and MSA α-syn strains are able to induce neurological deficits or neurodegeneration *in vitro* or *in vivo*. However, MSA-derived fibrils seem to have a stronger toxic potential (61, 72–74, 83).	• Use of different *in vivo* and *in vitro* models between studies. • Use of different genetic backgrounds (overexpression of WT or mutant forms of α-syn VS non-transgenic models).
Aggregation potential	PD, DLB and MSA α-syn strains are able to induce aggregation of endogenous α-syn *in vitro* and *in vivo* with MSA-derived α-syn showing higher aggregation potential (74–78, 83).	• Use of different sources of human α-syn (brain regions, age, sex, α-syn content).
Biochemical properties	MSA, PD and DLB strains present different proteinase K resistance and sarkosyl solubility patterns with MSA fibrils showing the highest resistance to protein degradation and DLB – the lowest (77, 78, 81, 83).	• Use of non-standardized protocols among studies or different experimental techniques.
Structural differences	MSA and PD fibrils exhibit twisted appearance, with MSA fibrils shoing higher twist frequency than PD fibrils. DLB fibrils are predominantly thinner with no twists (77, 81–83).	• Small number of cases included. • Limited number of studies including all α-synucleinopathies.
Amplification kinetics	MSA strains exhibit different amplification kinetics compared to PD and DLB strains in PMCA and RT-QuIC studies (99–101).	

Masuda-Suzukake and colleagues were the first ones to prove that the nigral injection of insoluble α-syn from human DLB brains in wt mice elicits α-syn pathology in various brain areas resembling mostly Lewy neurite-like structures (61). Notably, only 50% of the inoculated mice developed α-syn deposits and the ones who did, lacked neurological deficits (61). Moreover, DLB-derived α-syn showed lower propagation efficiency than PFFs, which could be explained by a lower concentration of α-syn in the brain extracts or reduced pathogenicity of DLB homogenates (61). Following Masuda-Suzukake's study, in the same year, Watts and colleagues tried a similar approach to investigate the transmission of MSA-derived α-syn (72). In their studies, they injected brain homogenates from MSA patients in hemizygous transgenic TgM83 mice, which overexpress human α-syn carrying the A53T mutation, inducing a rapidly progressive α-synucleinopathy in all animals that was accompanied by the emergence of neurological signs, such as ataxia and circling behavior (72). However, MSA-inoculated mice did not show α-syn deposition in oligodendrocytes, one of the major hallmarks of MSA (72). Only one year after the studies of Masuda-Suzukake and Prusiner, Recasens and colleagues injected LB-enriched fractions from PD brains in the substantia nigra and the striatum of wt mice and monkeys, observing in both the pathological conversion of endogenous α-syn associated with progressive PD-like neurodegeneration (73). Later on, transmission analyses of PD and MSA brain homogenates revealed that PD brain homogenates, in contrast to those of MSA patients, failed to produce signs of neurological dysfunction in inoculated hemizygous TgM83 mice, suggesting that MSA-derived α-syn possesses higher infectivity and pathogenic potential (74). Moreover, MSA-derived α-syn also demonstrated to efficiently spread from the injection site to other brain regions including the contralateral hemisphere (75) and, in contrast to DLB extracts, induced a greater burden of α-syn pathology compared to the PFFs in TgM83 mice (76). However, the latter study showed that MSA extracts did not induce α-syn pathology in transgenic expressing wild type human α-syn nor in non-transgenic mice (76). More recent studies comparing MSA with DLB or PD-derived α-syn revealed, in addition to morphological and biochemical differences, the ability of these strains to induce distinct pathological forms of α-syn, propagation patterns and seeding activities (77, 78) (Table 1).

Given all these findings highlighting the different characteristics of α-syn in a disease-specific context, structural analyses lacked to further demonstrate the existence of distinct disease-specific strains. In this regard, recent studies have finally made possible to partially disclose the structure of the different disease-derived α-syn fibrils through super resolution techniques (79). Using fluorescent probes, nuclear magnetic resonance (NMR) spectroscopy and electron paramagnetic resonance Strohäcker and colleagues first found that brain-derived α-syn strains were structurally different to *in vitro* α-syn conformers, however, they did not observe structural differences between PD and MSA-derived α-syn (80). Eventually, cryo-electron microscopy (cryo-EM) of α-syn derived from PD and MSA patients revealed that MSA strains present a higher portion of β-sheet structures and a greater number of twists, being more toxic and more resistant to protease degradation compared to PD strains (81). Similarly, Schweighauser and colleagues recently demonstrated that α-synuclein filaments from MSA brains also differ from those of individuals with DLB (82). Further studies corroborated structural differences between PD, DLB and MSA α-syn strains and demonstrated the ability of MSA-derived fibrils to induce a more significant motor impairment, neurodegeneration, neuroinflammation and α-syn seeding and spreading, reflecting the more aggressive nature of this disease compared to other synucleinopathies (83). Nevertheless, the existence of distinct and sometimes contradictory observations among the studies conducted till date must be acknowledge and could be explained by several limitations, including the use of different experimental models, techniques, source of human α-syn (brain region, α-syn load, etc.) or the small number of cases included (Table 1).

What causes the generation of different strains and the accompanied properties remains poorly understood. Considering that α-syn inclusions form in different cell types, it has been proposed that distinct α-syn strains are generated by different intracellular milieus (84). In this regard, experimental data indicated that α-syn strains formed in the cellular environment of oligodendrocytes obtain a more compact structure and achieve higher potency in seeding aggregation (84). However, patient-derived MSA strains fail to induce sufficient α-syn accumulation in oligodendrocytes, even though α-syn assemblies were observed to be taken up by oligodendrocytes *in vitro* (51, 76, 85). Therefore, MSA could constitute a primary oligodendrogliopathy (86) in which multiple factors, including aging (87), epigenetic and environmental factors (88), might lead to oligodendroglial impairment and thus to α-syn accumulation. Supporting this hypothesis, a recent study showed that the oligodendroglial protein p25α stimulates folding of an α-syn strain capable of templating neuronal α-syn *in vivo* with enhanced neurodegenerative potential, leading to a more aggressive disease course (89). Hence, the formation of GCIs in MSA might not be only bound to the presence of pathological α-syn but also to a permissive environment, possibly provided by oligodendrocytes themselves (79, 90). Further studies will be necessary to elucidate the sequence of events triggering the different α-synucleinopathies and to reveal the molecular and cellular mechanism underlying the generation of their distinct α-syn strains.

### Clinical Relevance and Future Perspectives

As discussed before, differences in age of onset, clinical symptoms, α-syn inclusion morphology and neuropathological distribution of α-synucleinopathies might originate from distinct α-syn strains (Figure 1). At present, the fact that these disorders share many clinical features and overlapping symptoms makes hard to differentiate them, leading to misdiagnosis and definitive diagnosis only possible post mortem (91). Hence, α-syn strains might represent a promising biomarker to facilitate the diagnosis.

Protein Misfolding Cyclic Amplification (PMCA) and Quaking-Induced Conversion (QuIC) are amplification techniques originally used to multiply prions that utilizes the ability of α-syn to self-propagate (92). Thus, small amounts of proteins can be increased to detectable concentrations by undergoing incubation cycles with excess of monomeric α-syn (92), which is especially useful based on the fact that protein misfolding and aggregation might occur decades before disease onset (93). In this regard, α-syn aggregates can be found not only in the brain but also in smaller amounts in peripheral tissues such as skin, olfactory mucosa, salivary glands, and gastrointestinal tract and in body fluids including cerebrospinal fluid (CSF) and blood (94). The presence of extracellular α-syn in CSF seems to derive from the CNS rather than from peripheral blood (95) and might be originated, among other mechanism, through the release of exosomes by neurons, astrocytes, microglia, and oligodendrocytes (96). These small vesicles of endosomal origin can carry a plethora of proteins into the CSF or the periphery, including small amounts of disease-specific and amyloidogenic proteins such as pathogenic α-syn (96, 97). Thus, recent studies have demonstrated the ability of PMCA and QuIC to detect α-syn aggregates with high sensitivity and specificity in the CSF of α-synucleinopathy patients, successfully differentiating PD, DLB and MSA strains by measuring the kinetics of aggregation in real-time through the binding of the fluorescent signal thioflavin T, which specifically binds amyloid fibrils (81, 83, 98–100). These differential amplification patterns are believed to rely on the unique structural and biochemical properties of the different strains, which are maintained after amplification (81). Interestingly, a couple of recent studies demonstrated the existence of significantly different amplification kinetics of CSF-derived α-syn from MSA patients compared to PD and DLB, that is already present in early clinically diagnosed MSA patients (99, 100). Hence, these findings support the use of amplification methods to differentiate MSA from PD and DLB even at early stages. Unfortunately, PD and DLB are harder to distinguish by this technique having very similar patterns (99, 100). However, experimental data indicate that these two latter strains differ as demonstrated by transmission electron microscopy and protein kinase digestion (89). Similar to biochemical, functional and structural studies, the use of different amplification protocols and source of human α-syn might be an important limiting factor of current research. Therefore, the use of standardized methods in future analyses should be considered.

Recent studies have also tried to decipher whether α-syn strains from PAF or iRBD patients differ from the ones of PD, DLB and MSA. In this regard, Singer and colleagues demonstrated the presence of α-syn in the CSF of most PAF cases included in the study, showing a similar amplification kinetic to PD and DLB cases, and significantly different from those with MSA (101). In addition, they observed that all PAF patients that later progressed toward MSA were the only ones showing amplification kinetics previously described in MSA patients (101). Thus, the use of amplification assays might constitute a potential diagnostic tool for MSA even at early-stages. As for iRBD, Iranzo and colleagues recently confirmed the presence of α-syn in the CSF from most iRBD cases (90%) after PMCA analysis (102). However, they did not detect different amplification kinetics between iRBD cases that converted to PD or DLB and those who did not convert to any other α-synucleinopathy (102). Another recent study by Stefani and colleagues demonstrated that amplification analysis enables the molecular detection of α-syn in the olfactory mucosa of patients with iRBD and PD, although with less sensitivity compared to CSF-based studies (103). Similarly to CSF, no differences in amplification kinetics were observed between iRBD and PD olfactory biopsies (103). Further studies including MSA cases should be conducted to clarify whether the use of this technique could be helpful to isolate potential MSA cases from other iRBD cases at early-stages. Moreover, it remains to be determined whether PAF and iRBD are associated with the presence of specific and distinct α-syn strains, or if they simply constitute early phases of the classical α-synucleinopathies (Figure 1).

It has to be mentioned that, till date, most amplification studies have been based on the use of CSF, which represents an invasive approach that might be difficult to introduce in preventive screenings. Therefore, it would be favorable to substitute CSF by a more accessible biological fluid, such as blood. In this regard, α-syn oligomers have been detected in the plasma of PD patients, but it remains to be determined if their presence precedes neuronal loss (104). A recent study by Dutta and colleagues confirmed the presence of α-syn in the blood of patients by exosome immunoprecipitation using neuronal and oligodendroglial markers, observing significantly higher α-syn concentrations in MSA exosomes compared to PD and healthy controls (105). The use of skin biopsies containing autonomic nerve terminals has also been suggested as a potential alternative source to investigate the seeding capability of α-syn, with comparable sensitivity and specificity to CSF (106). However, in these latter studies, samples were either obtained post-mortem or from clinically diagnosed patients, therefore future amplification studies should evaluate α-syn seeding capability in peripheral tissues and biological fluids at early stages. Thus, although the presence of α-syn pathological species often occurs years before symptoms become evident, it has yet to be established at which time point they are detectable and distinguishable from control cases and where (107).

In summary, the complete characterization of the presence of pathological α-syn throughout disease progression in biological fluids, skin or peripheral biopsies combined with the use of amplification assays, super resolution techniques and biochemical analyses, constitute a promising strategy to differentiate α-synucleinopathies. This might allow clinicians to diagnose the different α-synucleinopathies even at early stages, facilitating the recruitment of proper patient cohorts for future clinical trials. In this regard, molecules inhibiting the seeding and spreading of α-syn pathological species represent novel and potential therapeutic strategies for disease modification (2).

## Conclusion

α-synucleinopathies constitute an important group of neurodegenerative disorders, some of them leading to death a few years after symptom-onset, with no treatment available. Although they share different clinical and pathological features, it is unclear what triggers these disorders and which mechanisms are responsible for their different neuropathological patterns. Recent findings indicate the existence of distinct α-syn strains with different structural and kinetic properties that could explain the distinctive pathological features, disease durations and severity of α-synucleinopathies. Despite these observations, how the unique structural and biological characteristics of α-syn strains might underlie disease heterogeneity must be further elucidated. Nevertheless, the existence of different α-syn strains and their identification represents a novel and promising diagnostic tool to differentiate these disorders, even at early stages, and constitute a potential prognostic biomarker for the development of early disease interventions.

## Author Contributions

AHG contributed to conception and design of the mini-review. KM wrote the first draft of the manuscript. NS and AHG wrote sections of the manuscript. All authors contributed to manuscript revision, read, and approved the submitted version.

## Funding

This work was supported by a grant of the Austrian Science Funds (FWF) F4414.

## Conflict of Interest

The authors declare that the research was conducted in the absence of any commercial or financial relationships that could be construed as a potential conflict of interest.

## Publisher's Note

All claims expressed in this article are solely those of the authors and do not necessarily represent those of their affiliated organizations, or those of the publisher, the editors and the reviewers. Any product that may be evaluated in this article, or claim that may be made by its manufacturer, is not guaranteed or endorsed by the publisher.

## Abbreviations

α-syn: α-synuclein
CNS: central nervous system
CSF: cerebrospinal fluid
DLB: dementis with Lewy bodies
GCIs: glial cytoplasmic inclusions
iRBD: isolated rapid eye movement sleep behavior disorder
MSA: multiple system atrophy
NAC: non-amyloid component
LBs: Lewy bodies
OPCA: olivopontocerebellar atrophy
PAF: pure autonomic failure
PD: Parkinson's disease
PFFs: α-syn pre-formed fibrils
PMCA: protein misfolding cyclic amplification
SND: striatonigral degeneration
WT: wild-type.

## References

[1] Lashuel HA Overk CR Oueslati A Masliah E. The many faces of alpha-synuclein: from structure and toxicity to therapeutic target. Nat Rev Neurosci. (2013) 14:38–48. 10.1038/nrn3406 23254192 PMC4295774

[2] Heras-Garvin A Stefanova N. From synaptic protein to prion: the long and controversial journey of α-synuclein. Front Synaptic Neurosci. (2020) 12:584536. 10.3389/fnsyn.2020.584536 33071772 PMC7536368

[3] Spillantini MG Crowther RA Jakes R Cairns NJ Lantos PL Goedert M. Filamentous alpha-synuclein inclusions link multiple system atrophy with Parkinson's disease and dementia with Lewy bodies. Neurosci Lett. (1998) 251:205–8. 10.1016/S0304-3940(98)00504-7 9726379

[4] Delamarre A Meissner WG. Epidemiology, environmental risk factors and genetics of Parkinson's disease. Presse Med. (2017) 46:175–81. 10.1016/j.lpm.2017.01.001 28189372

[5] Jellinger KA. Dementia with Lewy bodies and Parkinson's disease-dementia: current concepts and controversies. J Neural Transm (Vienna). (2018) 125:615–50. 10.1007/s00702-017-1821-9 29222591

[6] Gallagher DA Lees AJ Schrag A. What are the most important nonmotor symptoms in patients with Parkinson's disease and are we missing them? Mov Disord. (2010) 25:2493–500. 10.1002/mds.23394 20922807

[7] Chu Y Dodiya H Aebischer P Olanow CW Kordower JH. Alterations in lysosomal and proteasomal markers in Parkinson's disease: relationship to alpha-synuclein inclusions. Neurobiol Dis. (2009) 35:385–98. 10.1016/j.nbd.2009.05.023 19505575

[8] Martinez-Vicente M Talloczy Z Kaushik S Massey AC Mazzulli J Mosharov EV. Dopamine-modified alpha-synuclein blocks chaperone-mediated autophagy. J Clin Invest. (2008) 118:777–88. 10.1172/JCI32806 18172548 PMC2157565

[9] Exner N Lutz AK Haass C Winklhofer KF. Mitochondrial dysfunction in Parkinson's disease: molecular mechanisms and pathophysiological consequences. EMBO J. (2012) 31:3038–62. 10.1038/emboj.2012.170 22735187 PMC3400019

[10] Funke C Schneider SA Berg D Kell DB. Genetics and iron in the systems biology of Parkinson's disease and some related disorders. Neurochem Int. (2013) 62:637–52. 10.1016/j.neuint.2012.11.015 23220386

[11] Hirsch EC Vyas S Hunot S. Neuroinflammation in Parkinson's disease. Parkinsonism Relat Disord. (2012) 18:S210–2. 10.1016/S1353-8020(11)70065-7 22166438

[12] McKeith IG Dickson DW Lowe J Emre M O'Brien JT Feldman H. Diagnosis and management of dementia with Lewy bodies: third report of the DLB consortium. Neurology. (2005) 65:1863–72. 10.1212/WNL.65.12.1992-a 16237129

[13] Outeiro TF Koss DJ Erskine D Walker L Kurzawa-Akanbi M Burn D. Dementia with Lewy bodies: an update and outlook. Mol Neurodegener. (2019) 14:5. 10.1186/s13024-019-0306-8 30665447 PMC6341685

[14] Walker L Stefanis L Attems J. Clinical and neuropathological differences between Parkinson's disease, Parkinson's disease dementia and dementia with Lewy bodies - current issues and future directions. J Neurochem. (2019) 150:467–74. 10.1111/jnc.14698 30892688

[15] Kaji S Maki T Ishimoto T Yamakado H Takahashi R. Insights into the pathogenesis of multiple system atrophy: focus on glial cytoplasmic inclusions. Transl Neurodegener. (2020) 9:7. 10.1186/s40035-020-0185-5 32095235 PMC7025408

[16] Krismer F Wenning GK. Multiple system atrophy: insights into a rare and debilitating movement disorder. Nat Rev Neurol. (2017) 13:232–43. 10.1038/nrneurol.2017.26 28303913

[17] Hoffmann A Ettle B Battis K Reiprich S Schlachetzki JCM Masliah E. Oligodendroglial alpha-synucleinopathy-driven neuroinflammation in multiple system atrophy. Brain Pathol. (2019) 29:380–96. 10.1111/bpa.12678 30444295 PMC6850330

[18] Vieira BD Radford RA Chung RS Guillemin GJ Pountney DL. Neuroinflammation in multiple system atrophy: response to and cause of alpha-synuclein aggregation. Front Cell Neurosci. (2015) 9:437. 10.3389/fncel.2015.00437 26778958 PMC4700780

[19] Kubler D Wachter T Cabanel N Su Z Turkheimer FE Dodel R. Widespread microglial activation in multiple system atrophy. Mov Disord. (2019) 34:564–8. 10.1002/mds.27620 30726574 PMC6659386

[20] Schwarz J Weis S Kraft E Tatsch K Bandmann O Mehraein P. Signal changes on MRI and increases in reactive microgliosis, astrogliosis, and iron in the putamen of two patients with multiple system atrophy. J Neurol Neurosurg Psychiatry. (1996) 60:98–101. 10.1136/jnnp.60.1.98 8558163 PMC486200

[21] Williams GP Marmion DJ Schonhoff AM Jurkuvenaite A Won WJ Standaert DG. T cell infiltration in both human multiple system atrophy and a novel mouse model of the disease. Acta Neuropathol. (2020) 139:855–74. 10.1007/s00401-020-02126-w 31993745 PMC7181566

[22] Fanciulli A Wenning GK. Multiple-system atrophy. N Engl J Med. (2015) 372:249–63. 10.1056/NEJMra1311488 25587949

[23] Seppi K Yekhlef F Diem A Luginger Wolf E Mueller J Tison F. Progression of parkinsonism in multiple system atrophy. J Neurol. (2005) 252:91–6. 10.1007/s00415-005-0617-2 15654560

[24] Jellinger KA. Multiple system atrophy: an oligodendroglioneural synucleinopathy1. J Alzheimers Dis. (2018) 62:1141–79. 10.3233/JAD-170397 28984582 PMC5870010

[25] Coon EA Singer W Low PA. Pure autonomic failure. Mayo Clin Proc. (2019) 94:2087–98. 10.1016/j.mayocp.2019.03.009 31515103 PMC6826339

[26] Hogl B Stefani A Videnovic A. Idiopathic REM sleep behaviour disorder and neurodegeneration - an update. Nat Rev Neurol. (2018) 14:40–55. 10.1038/nrneurol.2017.157 29170501

[27] Singer W Berini SE Sandroni P Fealey RD Coon EA Suarez MD. Pure autonomic failure: predictors of conversion to clinical CNS involvement. Neurology. (2017) 88:1129–36. 10.1212/WNL.0000000000003737 28202694 PMC5373781

[28] Kaufmann H Nahm K Purohit D Wolfe D. Autonomic failure as the initial presentation of Parkinson disease and dementia with Lewy bodies. Neurology. (2004) 63:1093–5. 10.1212/01.WNL.0000138500.73671.DC 15452307

[29] Coon EA Sletten DM Suarez MD Mandrekar JN Ahlskog JE Bower JH. Clinical features and autonomic testing predict survival in multiple system atrophy. Brain. (2015) 138:3623–31. 10.1093/brain/awv274 26369944 PMC4840547

[30] Kaufmann H Goldstein DS. Pure autonomic failure: a restricted Lewy body synucleinopathy or early Parkinson disease? Neurology. (2010) 74:536–7. 10.1212/WNL.0b013e3181d26982 20157156

[31] Park A Stacy M. Non-motor symptoms in Parkinson's disease. J Neurol. (2009) 256:293–8. 10.1007/s00415-009-5240-1 19711119

[32] Chan PC Lee HH Hong CT Hu CJ Wu D. REM Sleep Behavior Disorder (RBD) in Dementia with Lewy Bodies (DLB). Behav Neurol. (2018) 2018:9421098. 10.1155/2018/9421098 30018672 PMC6029467

[33] Barone DA Henchcliffe C. Rapid eye movement sleep behavior disorder and the link to alpha-synucleinopathies. Clin Neurophysiol. (2018) 129:1551–64. 10.1016/j.clinph.2018.05.003 29883833 PMC6495539

[34] Iranzo A Tolosa E Gelpi E Molinuevo JL Valldeoriola F Serradell M. Neurodegenerative disease status and post-mortem pathology in idiopathic rapid-eye-movement sleep behaviour disorder: an observational cohort study. Lancet Neurol. (2013) 12:443–53. 10.1016/S1474-4422(13)70056-5 23562390

[35] Schenck CH Boeve BF Mahowald MW. Delayed emergence of a parkinsonian disorder or dementia in 81% of older men initially diagnosed with idiopathic rapid eye movement sleep behavior disorder: a 16-year update on a previously reported series. Sleep Med. (2013) 14:744–8. 10.1016/j.sleep.2012.10.009 23347909

[36] Theillet FX Binolfi A Bekei B Martorana A Rose HM Stuiver M. Structural disorder of monomeric alpha-synuclein persists in mammalian cells. Nature. (2016) 530:45–50. 10.1038/nature16531 26808899

[37] Fauvet B Mbefo MK Fares MB Desobry C Michael S Ardah MT. alpha-Synuclein in central nervous system and from erythrocytes, mammalian cells, and Escherichia coli exists predominantly as disordered monomer. J Biol Chem. (2012) 287:15345–64. 10.1074/jbc.M111.318949 22315227 PMC3346117

[38] Bartels T Choi JG Selkoe DJ. alpha-Synuclein occurs physiologically as a helically folded tetramer that resists aggregation. Nature. (2011) 477:107–10. 10.1038/nature10324 21841800 PMC3166366

[39] Wang W Perovic I Chittuluru J Kaganovich A Nguyen LT Liao J. A soluble alpha-synuclein construct forms a dynamic tetramer. Proc Natl Acad Sci U S A. (2011) 108:17797–802. 10.1073/pnas.1113260108 22006323 PMC3203798

[40] Serpell LC Berriman J Jakes R Goedert M Crowther RA. Fiber diffraction of synthetic alpha-synuclein filaments shows amyloid-like cross-beta conformation. Proc Natl Acad Sci U S A. (2000) 97:4897–902. 10.1073/pnas.97.9.4897 10781096 PMC18329

[41] Celej MS Sarroukh R Goormaghtigh E Fidelio GD Ruysschaert JM Raussens V. Toxic prefibrillar alpha-synuclein amyloid oligomers adopt a distinctive antiparallel beta-sheet structure. Biochem J. (2012) 443:719–26. 10.1042/BJ20111924 22316405

[42] Roeters SJ Iyer A Pletikapic G Kogan V Subramaniam V Woutersen S. Evidence for intramolecular antiparallel beta-sheet structure in alpha-synuclein fibrils from a combination of two-dimensional infrared spectroscopy and atomic force microscopy. Sci Rep. (2017) 7:41051. 10.1038/srep41051 28112214 PMC5253669

[43] Tuttle MD Comellas G Nieuwkoop AJ Covell DJ Berthold DA Kloepper KD. Solid-state NMR structure of a pathogenic fibril of full-length human alpha-synuclein. Nat Struct Mol Biol. (2016) 23:409–15. 10.1038/nsmb.3194 27018801 PMC5034296

[44] Bertoncini CW Jung YS Fernandez CO Hoyer W Griesinger C Jovin TM. Release of long-range tertiary interactions potentiates aggregation of natively unstructured alpha-synuclein. Proc Natl Acad Sci U S A. (2005) 102:1430–5. 10.1073/pnas.0407146102 15671169 PMC547830

[45] Hijaz BA Volpicelli-Daley LA. Initiation and propagation of alpha-synuclein aggregation in the nervous system. Mol Neurodegener. (2020) 15:19. 10.1186/s13024-020-00368-6 32143659 PMC7060612

[46] Meade RM Fairlie DP Mason JM. Alpha-synuclein structure and Parkinson's disease - lessons and emerging principles. Mol Neurodegener. (2019) 14:29. 10.1186/s13024-019-0329-1 31331359 PMC6647174

[47] Djelloul M Holmqvist S Boza-Serrano A Azevedo C Yeung MS Goldwurm S. Alpha-synuclein expression in the oligodendrocyte lineage: an *in vitro* and *in vivo* study using rodent and human models. Stem Cell Reports. (2015) 5:174–84. 10.1016/j.stemcr.2015.07.002 26235891 PMC4618831

[48] Miller DW Johnson JM Solano SM Hollingsworth ZR Standaert DG Young AB. Absence of alpha-synuclein mRNA expression in normal and multiple system atrophy oligodendroglia. J Neural Transm (Vienna). (2005) 112:1613–24. 10.1007/s00702-005-0378-1 16284907

[49] Asi YT Simpson JE Heath PR Wharton SB Lees AJ Revesz T. Alpha-synuclein mRNA expression in oligodendrocytes in MSA. Glia. (2014) 62:964–70. 10.1002/glia.22653 24590631 PMC4238782

[50] Angot E Steiner JA Lema Tome CM Ekstrom P Mattsson B Bjorklund A. Alpha-synuclein cell-to-cell transfer and seeding in grafted dopaminergic neurons in vivo. PLoS ONE. (2012) 7:e39465. 10.1371/journal.pone.0039465 22737239 PMC3380846

[51] Reyes JF Rey NL Bousset L Melki R Brundin P Angot E. Alpha-synuclein transfers from neurons to oligodendrocytes. Glia. (2014) 62:387–98. 10.1002/glia.22611 24382629

[52] Kordower JH Chu Y Hauser RA Freeman TB Olanow CW. Lewy body-like pathology in long-term embryonic nigral transplants in Parkinson's disease. Nat Med. (2008) 14:504–6. 10.1038/nm1747 18391962

[53] Li JY Englund E Holton JL Soulet D Hagell P Lees AJ. Lewy bodies in grafted neurons in subjects with Parkinson's disease suggest host-to-graft disease propagation. Nat Med. (2008) 14:501–3. 10.1038/nm1746 18391963

[54] Delenclos M Burgess JD Lamprokostopoulou A Outeiro TF Vekrellis K McLean PJ. Cellular models of alpha-synuclein toxicity and aggregation. J Neurochem. (2019) 150:566–76. 10.1111/jnc.14806 31265132 PMC8864560

[55] Magen I Chesselet MF. Mouse models of cognitive deficits due to alpha-synuclein pathology. J Parkinsons Dis. (2011) 1:217–27. 10.3233/JPD-2011-11043 23939303

[56] Lee HJ Ricarte D Ortiz D Lee SJ. Models of multiple system atrophy. Exp Mol Med. (2019) 51:1–10. 10.1038/s12276-019-0346-8 31740682 PMC6861264

[57] Koprich JB Kalia LV Brotchie JM. Animal models of alpha-synucleinopathy for Parkinson disease drug development. Nat Rev Neurosci. (2017) 18:515–29. 10.1038/nrn.2017.75 28747776

[58] Duffy MF Collier TJ Patterson JR Kemp CJ Fischer DL Stoll AC. Quality over quantity: advantages of using alpha-synuclein preformed fibril triggered synucleinopathy to model idiopathic Parkinson's disease. Front Neurosci. (2018) 12:621. 10.3389/fnins.2018.00621 30233303 PMC6132025

[59] Dehay B Vila M Bezard E Brundin P Kordower JH. Alpha-synuclein propagation: new insights from animal models. Mov Disord. (2016) 31:161–8. 10.1002/mds.26370 26347034

[60] Chung HK Ho HA Perez-Acuna D Lee SJ. Modeling alpha-Synuclein Propagation with Preformed Fibril Injections. J Mov Disord. (2019) 12:139–51. 10.14802/jmd.19046 31556259 PMC6763716

[61] Masuda-Suzukake M Nonaka T Hosokawa M Oikawa T Arai T Akiyama H. Prion-like spreading of pathological alpha-synuclein in brain. Brain. (2013) 136:1128–38. 10.1093/brain/awt037 23466394 PMC3613715

[62] Luk KC Kehm V Carroll J Zhang B O'Brien P Trojanowski JQ. Pathological alpha-synuclein transmission initiates Parkinson-like neurodegeneration in nontransgenic mice. Science. (2012) 338:949–53. 10.1126/science.1227157 23161999 PMC3552321

[63] Rey NL George S Steiner JA Madaj Z Luk KC Trojanowski JQ. Spread of aggregates after olfactory bulb injection of alpha-synuclein fibrils is associated with early neuronal loss and is reduced long term. Acta Neuropathol. (2018) 135:65–83. 10.1007/s00401-017-1792-9 29209768 PMC5756266

[64] Sacino AN Brooks M Thomas MA McKinney AB Lee S Regenhardt RW. Intramuscular injection of alpha-synuclein induces CNS alpha-synuclein pathology and a rapid-onset motor phenotype in transgenic mice. Proc Natl Acad Sci U S A. (2014) 111:10732–7. 10.1073/pnas.1321785111 25002524 PMC4115570

[65] Breid S Bernis ME Babila JT Garza MC Wille H Tamguney G. Neuroinvasion of alpha-synuclein prionoids after intraperitoneal and intraglossal inoculation. J Virol. (2016) 90:9182–93. 10.1128/JVI.01399-16 27489279 PMC5044858

[66] Kim S Kwon SH Kam TI Panicker N Karuppagounder SS Lee S. Transneuronal propagation of pathologic alpha-synuclein from the gut to the brain models Parkinson's disease. Neuron. (2019) 103:627–41 e7. 10.1016/j.neuron.2019.05.035 31255487 PMC6706297

[67] Uemura N Yagi H Uemura MT Hatanaka Y Yamakado H Takahashi R. Inoculation of alpha-synuclein preformed fibrils into the mouse gastrointestinal tract induces Lewy body-like aggregates in the brainstem via the vagus nerve. Mol Neurodegener. (2018) 13:21. 10.1186/s13024-018-0257-5 29751824 PMC5948849

[68] Wang XJ Ma MM Zhou LB Jiang XY Hao MM Teng RKF. Autonomic ganglionic injection of alpha-synuclein fibrils as a model of pure autonomic failure alpha-synucleinopathy. Nat Commun. (2020) 11:934. 10.1038/s41467-019-14189-9 32071315 PMC7028908

[69] Bousset L Pieri L Ruiz-Arlandis G Gath J Jensen PH Habenstein B. Structural and functional characterization of two alpha-synuclein strains. Nat Commun. (2013) 4:2575. 10.1038/ncomms3575 24108358 PMC3826637

[70] Peelaerts W Bousset L Van der Perren A Moskalyuk A Pulizzi R Giugliano M. alpha-Synuclein strains cause distinct synucleinopathies after local and systemic administration. Nature. (2015) 522:340–4. 10.1038/nature14547 26061766

[71] Lau A So RWL Lau HHC Sang JC Ruiz-Riquelme A Fleck SC. alpha-Synuclein strains target distinct brain regions and cell types. Nat Neurosci. (2020) 23:21–31. 10.1038/s41593-019-0541-x 31792467 PMC6930851

[72] Watts JC Giles K Oehler A Middleton L Dexter DT Gentleman SM. Transmission of multiple system atrophy prions to transgenic mice. Proc Natl Acad Sci U S A. (2013) 110:19555–60. 10.1073/pnas.1318268110 24218576 PMC3845125

[73] Recasens A Dehay B Bove J Carballo-Carbajal I Dovero S Perez-Villalba A. Lewy body extracts from Parkinson disease brains trigger alpha-synuclein pathology and neurodegeneration in mice and monkeys. Ann Neurol. (2014) 75:351–62. 10.1002/ana.24066 24243558

[74] Prusiner SB Woerman AL Mordes DA Watts JC Rampersaud R Berry DB. Evidence for alpha-synuclein prions causing multiple system atrophy in humans with parkinsonism. Proc Natl Acad Sci U S A. (2015) 112:E5308–17. 10.1073/pnas.1514475112 26324905 PMC4586853

[75] Bernis ME Babila JT Breid S Wusten KA Wullner U Tamguney G. Prion-like propagation of human brain-derived alpha-synuclein in transgenic mice expressing human wild-type alpha-synuclein. Acta Neuropathol Commun. (2015) 3:75. 10.1186/s40478-015-0254-7 26612754 PMC4660655

[76] Dhillon JS Trejo-Lopez JA Riffe C Levites Y Sacino AN Borchelt DR. Comparative analyses of the in vivo induction and transmission of alpha-synuclein pathology in transgenic mice by MSA brain lysate and recombinant alpha-synuclein fibrils. Acta Neuropathol Commun. (2019) 7:80. 10.1186/s40478-019-0733-3 31109378 PMC6526622

[77] Tarutani A Arai T Murayama S Hisanaga SI Hasegawa M. Potent prion-like behaviors of pathogenic alpha-synuclein and evaluation of inactivation methods. Acta Neuropathol Commun. (2018) 6:29. 10.1186/s40478-018-0532-2 29669601 PMC5907316

[78] Yamasaki TR Holmes BB Furman JL Dhavale DD Su BW Song ES. Parkinson's disease and multiple system atrophy have distinct alpha-synuclein seed characteristics. J Biol Chem. (2019) 294:1045–58. 10.1074/jbc.RA118.004471 30478174 PMC6341389

[79] Holec SAM Woerman AL. Evidence of distinct alpha-synuclein strains underlying disease heterogeneity. Acta Neuropathol. (2021) 142:73–86. 10.1007/s00401-020-02163-5 32440702

[80] Strohaker T Jung BC Liou SH Fernandez CO Riedel D Becker S. Structural heterogeneity of alpha-synuclein fibrils amplified from patient brain extracts. Nat Commun. (2019) 10:5535. 10.1038/s41467-019-13564-w 31797870 PMC6893031

[81] Shahnawaz M Mukherjee A Pritzkow S Mendez N Rabadia P Liu X. Discriminating alpha-synuclein strains in Parkinson's disease and multiple system atrophy. Nature. (2020) 578:273–7. 10.1038/s41586-020-1984-7 32025029 PMC7066875

[82] Schweighauser M Shi Y Tarutani A Kametani F Murzin AG Ghetti B. Structures of alpha-synuclein filaments from multiple system atrophy. Nature. (2020) 585:464–9. 10.1038/s41586-020-2317-6 32461689 PMC7116528

[83] Van der Perren A Gelders G Fenyi A Bousset L Brito F Peelaerts W. The structural differences between patient-derived alpha-synuclein strains dictate characteristics of Parkinson's disease, multiple system atrophy and dementia with Lewy bodies. Acta Neuropathol. (2020) 139:977–1000. 10.1007/s00401-020-02157-3 32356200 PMC7244622

[84] Peng C Gathagan RJ Covell DJ Medellin C Stieber A Robinson JL. Cellular milieu imparts distinct pathological alpha-synuclein strains in alpha-synucleinopathies. Nature. (2018) 557:558–63. 10.1038/s41586-018-0104-4 29743672 PMC5970994

[85] Woerman AL Oehler A Kazmi SA Lee J Halliday GM Middleton LT. Multiple system atrophy prions retain strain specificity after serial propagation in two different Tg(SNCA^*^A53T) mouse lines. Acta Neuropathol. (2019) 137:437–54. 10.1007/s00401-019-01959-4 30690664 PMC6454887

[86] Wenning GK Stefanova N Jellinger KA Poewe W Schlossmacher MG. Multiple system atrophy: a primary oligodendrogliopathy. Ann Neurol. (2008) 64:239–46. 10.1002/ana.21465 18825660

[87] Hou Y Dan X Babbar M Wei Y Hasselbalch SG Croteau DL. Ageing as a risk factor for neurodegenerative disease. Nat Rev Neurol. (2019) 15:565–81. 10.1038/s41582-019-0244-7 31501588

[88] Sturm E Stefanova N. Multiple system atrophy: genetic or epigenetic? Exp Neurobiol. (2014) 23:277–91. 10.5607/en.2014.23.4.277 25548529 PMC4276800

[89] Ferreira N Gram H Sorrentino ZA Gregersen E Schmidt SI Reimer L. Multiple system atrophy-associated oligodendroglial protein p25alpha stimulates formation of novel alpha-synuclein strain with enhanced neurodegenerative potential. Acta Neuropathol. (2021) 142:87–115. 10.1007/s00401-021-02316-0 33978813 PMC8217051

[90] Stefanova N Wenning GK. Review: Multiple system atrophy: emerging targets for interventional therapies. Neuropathol Appl Neurobiol. (2016) 42:20–32. 10.1111/nan.12304 26785838 PMC4788141

[91] Koga S Aoki N Uitti RJ van Gerpen JA Cheshire WP Josephs KA. When DLB, PD, and PSP masquerade as MSA: an autopsy study of 134 patients. Neurology. (2015) 85:404–12. 10.1212/WNL.0000000000001807 26138942 PMC4534078

[92] Paciotti S Bellomo G Gatticchi L Parnetti L. Are we ready for detecting alpha-synuclein prone to aggregation in patients? The case of “protein-misfolding cyclic amplification” and “real-time quaking-induced conversion” as diagnostic tools, Front Neurol. (2018) 9:415. 10.3389/fneur.2018.00415 29928254 PMC5997809

[93] Shahnawaz M Tokuda T Waragai M Mendez N Ishii R Trenkwalder C. Development of a biochemical diagnosis of Parkinson disease by detection of alpha-synuclein misfolded aggregates in cerebrospinal fluid. JAMA Neurol. (2017) 74:163–72. 10.1001/jamaneurol.2016.4547 27918765

[94] Fayyad M Salim S Majbour N Erskine D Stoops E Mollenhauer B. Parkinson's disease biomarkers based on alpha-synuclein. J Neurochem. (2019) 150:626–36. 10.1111/jnc.14809 31265130

[95] Mollenhauer B Trautmann E Otte B Ng J Spreer A Lange P. alpha-Synuclein in human cerebrospinal fluid is principally derived from neurons of the central nervous system. J Neural Transm (Vienna). (2012) 119:739–46. 10.1007/s00702-012-0784-0 22426833 PMC3378837

[96] Hornung S Dutta S Bitan G. CNS-derived blood exosomes as a promising source of biomarkers: opportunities and challenges. Front Mol Neurosci. (2020) 13:38. 10.3389/fnmol.2020.00038 32265650 PMC7096580

[97] Stuendl A Kunadt M Kruse N Bartels C Moebius W Danzer KM. Induction of alpha-synuclein aggregate formation by CSF exosomes from patients with Parkinson's disease and dementia with Lewy bodies. Brain. (2016) 139:481–94. 10.1093/brain/awv346 26647156 PMC4805087

[98] Candelise N Schmitz M Llorens F Villar-Pique A Cramm M Thom T. Seeding variability of different alpha synuclein strains in synucleinopathies. Ann Neurol. (2019) 85:691–703. 10.1002/ana.25446 30805957

[99] Rossi M Candelise N Baiardi S Capellari S Giannini G Orru CD. Ultrasensitive RT-QuIC assay with high sensitivity and specificity for Lewy body-associated synucleinopathies. Acta Neuropathol. (2020) 140:49–62. 10.1007/s00401-020-02160-8 32342188 PMC7299922

[100] Singer W Schmeichel AM Shahnawaz M Schmelzer JD Boeve BF Sletten DM. Alpha-synuclein oligomers and neurofilament light chain in spinal fluid differentiate multiple system atrophy from lewy body synucleinopathies. Ann Neurol. (2020) 88:503–12. 10.1002/ana.25824 32557811 PMC7719613

[101] Singer W Schmeichel AM Shahnawaz M Schmelzer JD Sletten DM Gehrking TL. Alpha-synuclein oligomers and neurofilament light chain predict phenoconversion of pure autonomic failure. Ann Neurol. (2021) 89:1212–20. 10.1002/ana.26089 33881777 PMC8168720

[102] Iranzo A Fairfoul G Ayudhaya ACN Serradell M Gelpi E Vilaseca I. Detection of alpha-synuclein in CSF by RT-QuIC in patients with isolated rapid-eye-movement sleep behaviour disorder: a longitudinal observational study. Lancet Neurol. (2021) 20:203–12. 10.1016/S1474-4422(20)30449-X 33609478

[103] Stefani A Iranzo A Holzknecht E Perra D Bongianni M Gaig C. Alpha-synuclein seeds in olfactory mucosa of patients with isolated REM sleep behaviour disorder. Brain. (2021) 144:1118–26. 10.1093/brain/awab005 33855335

[104] El-Agnaf OM Salem SA Paleologou KE Curran MD Gibson MJ Court JA. Detection of oligomeric forms of alpha-synuclein protein in human plasma as a potential biomarker for Parkinson's disease. FASEB J. (2006) 20:419–25. 10.1096/fj.03-1449com 16507759

[105] Dutta S Hornung S Kruayatidee A Maina KN Del Rosario I Paul KC. alpha-Synuclein in blood exosomes immunoprecipitated using neuronal and oligodendroglial markers distinguishes Parkinson's disease from multiple system atrophy. Acta Neuropathol. (2021) 142:495–511. 10.1007/s00401-021-02324-0 33991233 PMC8357708

[106] Wang Z Becker K Donadio V Siedlak S Yuan J Rezaee M. Skin alpha-synuclein aggregation seeding activity as a novel biomarker for Parkinson disease. JAMA Neurol. (2020) 78:30–40. 10.1001/jamaneurol.2020.3311 32986090 PMC7522783

[107] Surguchov A. Analysis of protein conformational strains-A key for new diagnostic methods of human diseases. Int J Mol Sci. (2020) 21:2801. 10.3390/ijms21082801 32316500 PMC7215537

